# Experimental chronic hepatitis B infection of neonatal tree shrews (*Tupaia belangeri chinensis*): A model to study molecular causes for susceptibility and disease progression to chronic hepatitis in humans

**DOI:** 10.1186/1743-422X-9-170

**Published:** 2012-08-23

**Authors:** Qi Wang, Paul Schwarzenberger, Fang Yang, Jingjing Zhang, Jianjia Su, Chun Yang, Ji Cao, Chao Ou, Liang Liang, Junlin Shi, Fang Yang, Duoping Wang, Jia Wang, Xiaojuan Wang, Ping Ruan, Yuan Li

**Affiliations:** 1 Department of Experimental Pathology, Guangxi Cancer Institute (Guangxi Tumor Hospital), Nanning 530021, China; 2 Quantum Immunologics, Tampa, FL, USA

**Keywords:** Tree shrew (*Tupaia*), Hepatitis B virus, Chronic infection

## Abstract

**Background:**

Hepatitis B virus (HBV) infection continues to be an escalating global health problem. Feasible and effective animal models for HBV infection are the prerequisite for developing novel therapies for this disease. The tree shrew (*Tupaia*) is a small animal species evolutionary closely related to humans, and thus is permissive to certain human viral pathogens. Whether tree shrews could be chronically infected with HBV in vivo has been controversial for decades. Most published research has been reported on adult tree shrews, and only small numbers of HBV infected newborn tree shrews had been observed over short time periods. We investigated susceptibility of newborn tree shrews to experimental HBV infection as well as viral clearance over a protracted time period.

**Results:**

Forty-six newborn tree shrews were inoculated with the sera from HBV-infected patients or tree shrews. Serum and liver samples of the inoculated animals were periodically collected and analyzed using fluorescence quantitative polymerase chain reaction, enzyme-linked immunosorbent assay, Southern blot, and immunohistochemistry. Six tree shrews were confirmed and four were suspected as chronically HBV-infected for more than 48 (up to 228) weeks after inoculation, including three that had been inoculated with serum from a confirmed HBV-infected tree shrew.

**Conclusions:**

Outbred neonatal tree shrews can be long-term chronically infected with HBV at a frequency comparable to humans. The model resembles human disease where also a smaller proportion of infected individuals develop chronic HBV related disease. This model might enable genetic and immunologic investigations which would allow determination of underlying molecular causes favoring susceptibility for chronic HBV infection and disease establishment vs. viral clearance.

## Background

Chronic hepatitis B virus (HBV) infection is an important global health problem, and is endemic in Southeast Asia and Africa. Although effective vaccinations are available, it has proven to be impossible providing large segments of the population in endemic regions with it. Therefore, the infected population is steadily expanding. As a result, many chronically HBV-infected patients will progress to developing cirrhosis and hepatocellular carcinoma [1]. For discovery and development of novel therapies, it is pivotal to have suitable in vivo disease models. A significant obstacle in developing such animal models is the fact that susceptibility to HBV is rather specific to humans. Only very closely related model is that of non-human primates, such as chimpanzees, who are also susceptible. However, use of these primates on a larger scale would be cost prohibitive.

Various hepadnavirus are infectious to small animals such as woodchuck, ground squirrel, duck and others, but these viruses vary significantly from HBV in structure and properties. The tree shrew (*Tupaia*) is evolutionary closely related to humans [2,3]. Blum and his colleagues have successfully infected HBV in extracorporeal hepatocytes of tree shrews, and proved permissiveness and replication of HBV in hepatocytes of this small animal [4-6]. However, thus far, experimental data suggested that tree shrew in vivo can only be transiently infected with HBV, which would limit the capabilities of this model in regards of studying chronic hepatitis in humans. Similarly, the majority of acute infected humans will ultimately clear the infection, with only a smaller portion developing chronic hepatitis. However, it is exactly this population that also carries the highest risk for developing cirrhosis and liver cancer. Therefore, suitable and more economic models mimicking chronic human HBV infection would be of significant value. It is not fully understood what are the exact risk factors which ultimately determine failure of virus clearance after the acute infection, and what factors exactly trigger and support progression to a chronic infection.

Nevertheless, clinical observations show that newborns and infants are more susceptible to infection with HBV, as well as progression to chronic infection. Similarly, susceptibility of animals to hepadnavirus is also inversely age-related [7-9]. It has been speculated that this might be due to the immaturity of the immune system. Previously, we published preliminary data from a small pilot study in support of this speculation [10]. In order to further consolidate these data and to generate unequivocal scientific proof for this hypothesis, we continued the experiments with expanded animal cohorts. We investigated susceptibility and long-term progression of HBV-inoculated outbred, genetically heterogenous newborn tree shrews over a protracted time period up to 4 years.

## Results

### Neonatal HBV inoculation leads to a rate of 13% confirmed chronically infected tree shrews

Based on the predefined criteria, six out of the forty-six neonatally inoculated tree shrews were confirmed chronically HBV-infected. Their HBV-infection markers remained positive for significantly longer than 48 weeks (up to 228 weeks) post inoculation. In general, the chronically infected animals showed HBsAg in serum early and persistently, along with inconsistent detection of other markers (HBeAg, HBeAb and HBcAb). Their serum HBV DNA consistently ranged between 10^3^ and 10^5^ copies/ml after 12- or 24-weeks following inoculation, and HBV DNA recovered from liver biopsies ranged from 10^4^ to 10^8^ copies/μg liver DNA after 6- or 12-weeks following inoculation. Table 1 displays the time course for the infectious markers in all six confirmed chronically HBV-infected tree shrews (Table 1).

**Table 1:** Infectious marks of the six tree shrews confirmed as chronic-infection

**No.**	**Infection marker**	**Weeks after inoculation**							
		**12**	**24**	**36**	**48**	**60**	**72**	**175w**	**228w**
90-1	serum HBV DNA	/	/	/	/	/	/	5.10 × 10^3^	4.00 × 10^4^
	liver HBV DNA	1.09 × 10^6^	1.41 × 10^6^	8.05 × 10^5^	/	/	/	5.52 × 10^7^	1.71 × 10^8^
	HBsAg	/	/	/	/	/	/	495.50	628.99
	HBsAb	/	/	/	/	/	/	-	-
	HBeAg	/	/	/	/	/	/	+	-
	HBeAb	/	/	/	/	/	/	-	-
	HBcAb	/	/	/	/	/	/	-	-
97-1	serum HBV DNA	-	5.10 × 10^4^	1.20 × 10^4^	6.70 × 10^3^	5.64 × 10^4^	1.40 × 10^4^		
	liver HBV DNA	1.70 × 10^5^	2.50 × 10^7^	3.10 × 10^7^	4.40 × 10^7^	1.08 × 10^8^	8.89 × 10^8^		
	HBsAg	/	/	/	/	/	281.60		
	HBsAb	/	/	/	/	/	-		
	HBeAg	/	/	/	/	/	-		
	HBeAb	/	/	/	/	/	+		
	HBcAb	/	/	/	/	/	+		
98-2	serum HBV DNA	-	3.10 × 10^4^	7.10 × 10^4^	4.01 × 10^2^	2.81 × 10^4^	9.48 × 10^2^		
	liver HBV DNA	3.50 × 10^6^	1.00 × 10^7^	2.30 × 10^7^	2.36 × 10^7^	4.26 × 10^6^	1.53 × 10^7^		
	HBsAg	/	/	/	/	/	380.00		
	HBsAb	/	/	/	/	/	-		
	HBeAg	/	/	/	/	/	+		
	HBeAb	/	/	/	/	/	-		
	HBcAb	/	/	/	/	/	+		
121-1	serum HBV DNA	8.74 × 10^2^	3.04 × 10^2^	1.24 × 10^3^	9.8 × 10^3^	7.37 × 10^1^	4.99 × 10^5^		
	liver HBV DNA	1.61 × 10^4^	6.88 × 10^7^	8.13 × 10^8^	2.50 × 10^7^	5.37 × 10^6^	2.68 × 10^8^		
	HBsAg	0.14	111.97	424.00	334.12	260.92	380.99		
	HBsAb	-	-	-	-	-	-		
	HBeAg	-	+	+	+	-	+		
	HBeAb	-	-	-	-	-	-		
	HBcAb	-	-	+	+	+	+		
122-1	serum HBV DNA	1.11 × 10^3^	-	-	9.68 × 10^2^	2.06 × 10^2^	3.73 × 10^5^		
	liver HBV DNA	3.05 × 10^4^	7.12 × 10^3^	1.27 × 10^4^	3.23 × 10^4^	1.10 × 10^7^	1.82 × 10^8^		
	HBsAg	0.18	-	-	-	3.78	-		
	HBsAb	+	±	±	-	-	-		
	HBeAg	-	-	-	-	-	-		
	HBeAb	-	-	-	-	-	-		
	HBcAb	-	-	-	-	-	-		
123-3	serum HBV DNA	-	1.29 × 10^3^	-	8.46 × 10^2^	3.63 × 10^3^	6.56 × 10^5^		
	liver HBV DNA	6.36 × 10^4^	3.07 × 10^4^	4.19 × 10^4^	1.12 × 10^5^	1.92 × 10^8^	1.16 × 10^8^		
	HBsAg	±	11.47	3.47	+	+	395.43		
	HBsAb	-	-	-	-	-	-		
	HBeAg	±	-	-	-	-	+		
	HBeAb	-	-	-	-	-	-		
	HBcAb	-	-	-	-	+			

HBV DNA in serum and liver samples was detected by FQ-PCR, the result of HBV DNA in serum was expressed as copy/ml, in liver was expressed as copy/μg liver DNA. Serum HBsAg was detected by ELISA and TRFIA, while HBsAb, HBeAg, HBeAb and HBcAb were detected by ELISA only. The results of ELISA were expressed as “+” (positive), “-” (negative) or “±” (between positive and negative). The result of TRFIA was expressed as ng/ml. “/” Indicated there was no test at that time point.

Four animals were suspected as chronically HBV-infected, because they showed intermittently weak-positive serum HBsAg and low level of HBV DNA in serum and/or in liver beyond 48-week post inoculation. Serum HBsAb in these animals remained continuously negative or was only occasionally positive at the early time after inoculation. Table 2 shows the details (Table 2).

**Table 2:** Infectious marks of the four tree shrews suspected as chronic-infection*

**Animal No.**	**HBV-infection marker**	**Weeks after inoculation**					
		**12w**	**24w**	**36w**	**48w**	**60w**	**72w or later**
117	serum HBV DNA	-	-	-	-	/	1.4 × 10^3^
	liver HBV DNA	-	-	-	-	/	3.3 × 10^4^
	HBsAg	0.17	-	-	-	/	0.09
	HBsAb	+	-	±−	-	/	-
121-2	serum HBV DNA	-	-	-	4.1 × 10^3^	/	/
	liver HBV DNA	-	-	8.2 × 10^4^	4.1 × 10^4^	/	/
	HBsAg	0.3	-	-	-	/	/
	HBsAb	-	±	-	-	/	/
122-2	serum HBV DNA	-	-	-	-	-	-
	liver HBV DNA	1.1 × 10^5^	1.9 × 10^4^	-	-	-	7.3 × 10^4^
	HBsAg	0.23	-	-	-	-	-
	HBsAb	+	±	±	-	-	-
140-3	serum HBV DNA	-	-	/	-	/	/
	liver HBV DNA	3.4 × 10^4^	3.1 × 10^4^	/	5.9 × 10^4^	/	/
	HBsAg	-	-	/	-	/	/
	HBsAb	+	-	/	-	/	/
	HBcAb	+	-	/	-	/	/

*: The criteria for the animals suspected of persistent infection was as following: The animals that up to the last time of tests (48 weeks or longer after inoculation), showed more than twice weakly positive HBsAg in serum, HBV DNA copy number ≥10^3^ in serum or ≥ 10^4^ in liver tissue. Meanwhile, their serum HBsAb were negative continuously, or were only occasionally positive at the early stage after inoculation.

### Isolation of genomic HBV DNA at various replication stages from liver tissue of infected tree shrews

**Figure 1:**
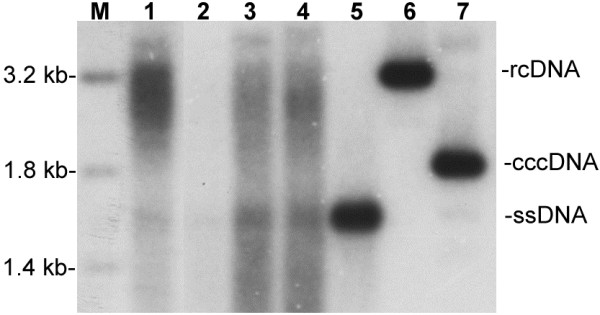
**Result of Southern blot.** Figure showed various intermediates of HBV DNA such as ssDNA (single strand DNA) and rcDNA (relaxed circular DNA) in liver biopsies from two of the chronically HBV-infected tree shrews collected at different time points after inoculation. The amount of HBV replication intermediates in tree shrew no. 98-2 showed a growing trend with time, though the form of HBV cccDNA (covalently closed circular DNA) was not confirmed well. M: Linear markers. 1: Liver samples of animal no. 90-1 collected at the 18^th^-week after inoculation. 2-4: Liver samples of animal no. 98-2 collected at the 12^th^-, 24^th^- and 36^th^-week after inoculation, respectively. 5: ssDNA, obtained by denaturing HBV rcDNA at 95°C for 5 min. 6: rcDNA, extracted from HBV-patient’s serum. 7: cccDNA size marker, a circular plasmid with 3.2 kb.

Southern blot analysis was performed on liver biopsies obtained from neonatally infected tree shrews at 12–36 weeks after inoculation. Different forms of HBV DNA (ssDNA and rcDNA) were identified, although HBV cccDNA could not definitely confirmed (Figure 1).

**Figure 2:**
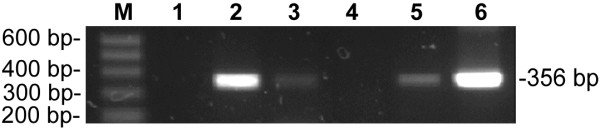
**HBV cccDNA in liver detected by nPCR.** M: Molecular size standard, 100 bp ladder. 1: Negative control, sterile water. 2-5: Liver biopsies of tree shrews. Lanes 2, 3 and 5 showed positive bands at 356 bp. These three positive samples were collected from animal no. 90-1 at 2 years and 3 moths, no. 97-1 at 48 weeks and no. 98-2 at 42 weeks after inoculation, respectively. Sequence analysis showed they correspond to the sequence of HBV. 6: Positive control of HBV cccDNA, recombinant plasmid pUC18-HBV.

HBV cccDNA was identified however in three of the chronically infected tree shrews by nPCR. Presence of HBV cccDNA was further confirmed by sequence analysis of the nucleotide bands with 356 bp isolated from the gel (data not shown) (Figure 2).

### Chronically HBV-infected tree shrews demonstrates hepatocellular HBV in situ and development of histopathologic changes

**Figure 3:**
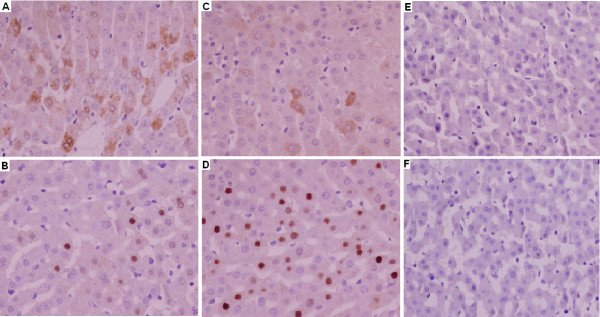
**Immunohistochemical detection of HBsAg and HBcAg in tree shrew’s liver biopsies, DAB staining, ×400.A** and **B**: Liver samples of animal no. 90-1 collected at the 167^th^-week after inoculation. A showed HBsAg-positive hepatocytes and B showed HBcAg-positive hepatocytes. **C** and **D**: Liver samples of animal no. 121-1 collected at the 60^th^-week after inoculation. C showed HBsAg-positive hepatocytes and D showed HBcAg-positive hepatocytes. **E** and **F**: Liver samples from an age-matched uninfected tree shrew. E showed HBsAg-negative hepatocytes and F showed HBcAg-negative hepatocytes.

Immunohistochemical staining on liver biopsies of all six chronically infected animals showed HBsAg- or HBcAg-positive hepatocytes. HBsAg-positive hepatocytes were observed as early as 24-week after inoculation. Figure 3 shows immunohistochemically stained representative sections. No staining was observed in liver tissue from uninfected tree shrews (Figure 3).

**Figure 4:**
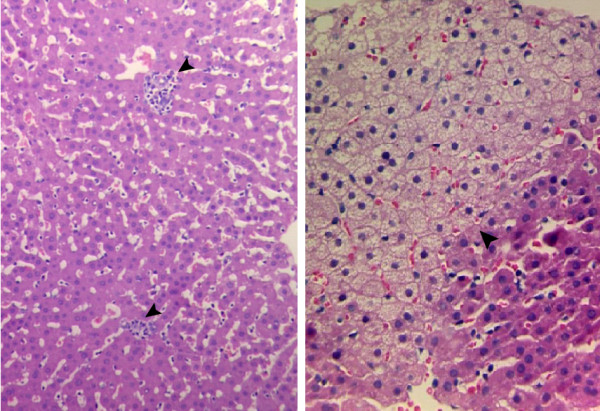
**Histopathological changes in liver of tree shrew with chronic HBV-infection.** Tree shrews were inoculated with HBV and sequential liver biopsies were obtained. HE-stained liver biopsies from representative chronically HBV-infected animals are depicted. Left: Biopsy was collected from animal 90-1 at 2.5 years post HBV inoculation and shows scattered or focally accumulated inflammatory mononuclear cells (arrows), ×200. Right: Biopsy was collected from animal 97-1 at 48 weeks post HBV inoculation and shows a proliferative focus (arrow) which is composed of transparent hepatocytes. ×400.

Histological examination of liver biopsies from six persistently infected tree shrews revealed relatively mild, although significant pathological changes, such as hepatocellular proliferation, degeneration, suspicious apoptosis and infiltration of chronic inflammatory cells. No obvious fibrosis was observed (Figure 4).

### Preservation of HBV genotype and infectivity throughout life cycle in tree shrews

Genotype analysis of HBV recovered from chronically infected tree shrews at different time point demonstrated consistency and preservation of the original genotype of the HBV used for infection.

Two sources of inocula were used to infect tree shrews, serum from HBV-infected human and serum from HBV-infected tree shrew. No difference between the infection rates of either inoculum was detected (Chi-Square test analysis, χ^2^ = 0.24, P = 0.624). Of nine animals inoculated with HBV-infected tree shrew (90–1) serum, one (121–1) was confirmed and two (117 and 121–2) were suspected of having chronic infection. These data demonstrate presence of replication-competent virus in tree shrews, as well as the infectious capability of HBV among tree shrews (Table 3).

**Table 3:** Incidence of chronic infection

**Inoculum**	No*	**Confirmed chronic infection (%)**	**Suspected chronic- infection**	**Total chronic- infection (%)**
Serum from HBV-patient	37	5 (13.5)	2	7 (18.9)
Serum from HBV-infected tree shrew	9	1 (11.1)	2	3 (33.3)
Total	46	6 (13.0)	4	10 (21.7)

*: Number of inoculated animal.

Analyzed by Chi-Square test with SPSS 13.0 statistics software, the infection incidence of the two sources of inoculum (serum of human and serum of tree shrew) were not significantly different (χ^2^ = 0.24, P = 0.624).

## Discussion

In this study we provide evidence that 13-21% of outbred, genetically heterogenous newborn tree shrews are susceptible to chronic HBV infection lasting at least 48 weeks (up to 228 weeks). Given a life expectancy for tree shrews of about 6–8 years, we arbitrarily set the time duration of 48 weeks as criterion defining chronic HBV-infection. The data presented demonstrate that HBV is capable of sustaining itself at a viable and replication competent infectious state in tree shrews with formation of intermediate replication products over prolonged periods.

Development of chronic hepatitis in tree shrews demonstrates other epidemiologic similarities such as age-related susceptibility to infection with its human counterpart. We failed to inoculate adult tree shrews (data not shown), but similar to human neonates who also are significantly more susceptible to HBV infection, we achieved persistent infection in a smaller portion of neonate animals.

In contrast to a genetically homogeneous animal strain, an outbred population allows to investigate genetic and immunologic heterogenic predispositions for chronic HBV infection susceptibility, disease establishment and progression. Therefore, the described model would be an economic research tool which could facilitate identification of individuals at risk for infection and also aid in the development of new therapies. However, this would also require full understanding of tree shrew biology and generation of standardized research reagents. In this context, full sequencing of the tree shrew genome and allocation of appropriate resources might be considered by governmental or private agencies.

A direct relationship between intrahepatic HBV DNA and clinical outcome parameters had been previously reported [11-13]. Although the copy numbers of serum HBV DNA in our study were generally lower when compared to humans suffering from chronic HBV-infection, we consistently confirmed presence of hepatic HBV DNA prior to its detection in serum in animals later to be confirmed as chronically infected. Namely, HBV copy number was higher in livers but lower in sera of tree shrews comparing to the values from human control samples. These data indicate that the liver is the main replication site of HBV in tree shrews, which precedes the appearance of detectable virus in serum. Therefore, we view the examination of liver biopsies as an important tool in order to predicting potential establishment of chronic HBV infection in tree shrews. Interestingly, we identified a significant number of tree shrews that maintained a persistent low burden of liver HBV DNA (10^3^-10^4^ copies/μg liver DNA) and negative HBsAg. This presentation closely resembles a clinical condition known as “occult HBV infection” in humans. Because occult HBV infection carries a high risk of developing chronic liver diseases including cancer in humans [14,15], this model could provide a unique opportunity studying this condition further in vivo.

Histopathological changes were relatively mild, even in liver biopsies from confirmed long-term HBV-infected tree shrews. Possible explanations might be the relatively short observation period, although it still constituted a significant portion of the entire life span of a tree shrew. Moreover, the obtainable tissue specimens were extremely small and thus accurate assessment of histopathologically observed changes is rather limited and not entirely transferable to the entire organ. Interestingly, a similar situation (lack of chronic liver histopathologic changes compared to human pathology) is also seen within other animal models of hepatitis virus infection, such as the woodchuck model of woodchuck hepatitis B virus (WHBV) infection, and even the non-human primate chimpanzees models of HBV infection [16]. The reasons for the disparity of histopathological changes between humans and animals chronically infected with hepatitis virus remains unknown.

Regarding the factors affecting HBV infectivity, some points should be considered. For example, when using human HBV-infected serum to inoculate primary tupaia hepatocytes (PTH), Cock et al. [5] demonstrated that serum purification increased infectivity and this was explained with the supposition that human serum could inhibit binding of HBV virions to PTH. Meanwhile, various studies indicate that high titer of HBV DNA and/or certain HBV genotypes may associated with an increased risk of developing chronic liver disease in humans [17-19]. Cote et al. [7] showed that besides age, the dose of virus inoculum to be a major factor influencing the course of chronic infection in woodchucks. In our study, the rate of chronic-infection induced by sera derived from HBV-infected tupaia appears to be higher than that by sera derived from HBV-infected humans, although the sample number is too small to make reliable statements concerning the significance of these differences. However, when comparing tree shrews inoculated with sera from HBV-infected humans to tree shrews inoculated with sera from HBV-infected tree shrews, the latter usually displayed lower levels of HBsAb, or their HBsAb appeared only intermittently. As an immunologic marker, appearance of HBsAb indicates clinical resolution of the HBV infection. In this context, this observation could suggest that using sera from the same infected species for inoculation might cause a lesser immune response than using infected serum from another species for that purpose. Thus, using tupaia-derived HBV-infected serum for inoculation might improve the infection rate. This hypothesis is currently being further investigated in our laboratory.

Although the frequency of infections in our model might resemble certain real life scenarios, higher infection rates might be desirable for certain experimental settings. To accomplish this, we are currently investigating various approaches such as using HBV strains with higher pathogenicity for inoculation, purifying or concentrating the inoculation serum in order to achieve higher viral titer or infection efficiency.

## Conclusions

In summary, our data demonstrate that the tree shrew model has promise in studying pathophysiology of chronic HBV-infection, thus justifies further investments into studying the genome and biology of tree shrews in an effort to further consolidate it as a model for human disease.

## Materials and methods

### Animal experiments, sample collections and statistics

Animal experiments were carried out in accordance with the guidelines for care and use of laboratory animals issued by Chinese government. Animals were housed at the Laboratory Animal Center of Guangxi Medical University under monitoring of veterinarians. Details have been reported previously [20].

The founders of the tree shrews used in this study were derived from a population of wild tree shrews (*Tupaia belangeri chinensis*) originating from the Kunming Institute of Zoology, Chinese Academy of Science (Yunnan, China). These animals were genetically inhomogeneous and continued to be bred in our laboratory for years. Newborn descendants from this population were utilized for experiments.

Newborn tree shrews were injected with 0.3 ml HBV inoculum twice subcutaneously, on the first and third day after birth, respectively. Six weeks after inoculation, blood samples were collected once every 4–6 weeks, liver samples were collected every 6–12 weeks using anesthesia (1% pentobarbital and ketamine hydrochloride). Animals were observed for at least 24 weeks after inoculation, or observed continuously as long as HBV-infection marker could be detected. A portion of each serum sample was tested immediately for HBV immunological markers, including HBV surface antigen (HBsAg), HBV surface antibody (HBsAb), HBV e antigen (HBeAg), HBV e antibody (HBeAb) and HBV core antibody (HBcAb) by enzyme linked immunosorbent assay (ELISA), or further by time-resolved immunofluorescence analysis (TRFIA). The other portion of serum sample was stored at −80°C for later molecular tests. A piece of each liver biopsy sample was frozen immediately by immersion in liquid nitrogen followed by storing at −80°C, and the remaining was fixed within 10% (v/v) neutral formalin and then paraffin embedded.

Infection rates of the two inocula, i.e. serum collected from human HBV-carriers and serum from a HBV-infected tree shrew, were analyzed by Chi-Square test with SPSS 13.0 statistics software (IBM, Chicago, USA).

### Inocula and positive controls

Inocula for injecting the experimental tree shrews included serum collected from human HBV-carriers and serum from an infected tree shrew. The HBV DNA copy number was ≥10^7^/ml in human serum and 10^4^–10^5^ /ml in tree shrew serum, which was validated prior injection.

Positive control samples included human sera from chronically HBV-infected patients, and liver tissues obtained from HBV-infected patients who had undergone surgical resections for hepatocellular carcinoma (HCC) at Guangxi Tumor Hospital of China.

The study protocol was approved by the Ethical Committee of Guangxi Tumor Hospital in accordance with the guidelines issued by Chinese government, which conforms to the ethical guidelines of the 1975 Declaration of Helsinki. Informed consents in writing were obtained from all participating patients.

### Detection of HBV immunological markers in serum by ELISA and TRFIA

Serum specimens of the experimental tree shrews were analyzed qualitatively by ELISA, for HBsAg, HBsAb, HBeAg, HBeAb and HBcAb. HBsAg positive specimens were further analyzed quantitatively by TRFIA.

Both tests of ELISA and TRFIA were carried out by the Clinical Laboratory Center of Guangxi Tumor Hospital (Nanning, China), using validated procedures for their clinical specimens. Kehua Bio-engineering (Shanghai, China) was the manufacturer for the ELISA kit, which was operated with an automatic processor (ML-FAME, AusBio Company, Bonaduz, Switzerland). TRFIA was conducted also by using a commercial kit (Xinbo Biotechnology, Suzhou, China) and an automated immunoassay system (Wallac AutoDELFIA, PerkinElmer Company, Turku, Finland).

### Detection of HBV DNA in serum and liver by fluorescence quantitative polymerase chain reaction (FQ-PCR)

Recovery and extraction of viral DNA from serum was performed according to the manufacturer’s instruction provided by a FQ-PCR kit (Kehua Bio-engineering Co. Shanghai, China) and a previously published procedure [21]. Total DNA extraction and HBV DNA detection from liver tissue were performed following the procedures described by Cacciola et al. [12]. Briefly, liver tissue was homogenized in digestion buffer that contains NaCl, Tris–HCl, sodium dodecyl sulphate and ethylenediaminetetraacetic acid, followed by digestion with proteinase K overnight, and extraction with phenol/chloroform/Isoamyl alcohol (25:24:1). The concentration of liver total DNA was determined using a spectrophotometer at 260 nm.

FQ-PCR amplification and analysis was carried out by the Clinical Laboratory Center of the First Hospital of Guangxi Medical University (Nanning, China), utilizing the same standard procedures that were implemented for their clinical samples. The amplification and analysis was performed on an ABI 7300 analyzer (Applied Biosystems, Foster City, CA, USA). The amplification conditions were 50°C 2 min, 94°C 2 min, followed by 40 cycles of 94°C 10 sec and 60°C 30 sec. Each round of amplification was accompanied by a calibration test with a serial of standards supplied within the FQ-PCR kit.

Per serum-derived HBV DNA was determined as copy/ml. Liver tissue-derived HBV DNA was calculated as copy/μg liver DNA [12]. According to the kit’s manufacturer, the threshold for determining positivity of serum samples had been as ≥10^3^ copies/ml. However, since no manufacturer’s critical threshold value had been recommended for liver tissue, we determined it as ≥10^4^ copies/μg liver DNA. This assumption was based on a series of limiting dilution experiments using positive and negative controls (data not shown).

### Detection of HBV DNA in liver by Southern blot

Southern blot analysis was performed in the laboratory of Professor Blum at Freiburg University of Germany, using the procedures previously described by his group [5]. Briefly, 20 μg of digested tree shrew liver DNA was separated on a 1.4% agarose gel, then transferred onto nylon membranes (Amersham Pharmacia Biotech, Piscataway, NJ, USA). Hybridization was performed in Roti-Hybri-Quick solution (Roth Chemikalien, Karlsruhe, Germany) using a ^32^P-labeled full-length sequence of wild-type HBV DNA probe.

### Detection of HBV cccDNA in liver by nested polymerase chain reaction (nPCR)

The detection was performed according to the protocol published by Singh et al. [22]. Briefly, DNA extracted from tree shrew liver tissue was digested with Plasmid-Safe^TM^ ATP-Dependent DNase (Epicentre Technologies, Madison, USA) to reduce the non-ccc HBV-DNA. DNA purification was then performed using a kit from Tiangen Biotech Co. (Beijing, China). Primers for amplification were synthesized by Sangon Biotech Co. (Shanghai, China). The sequences of outer primers were 5’-GCTTTGCTGCTCCATTTACAC-3’ (nt1013-nt1033) and 5’-TTCCGGAAGTGTTGATAAGAT–3’ (nt2335-nt2315), of inner primers were 5’-CCGACCACGGGTCGCACCTCTC-3’ (nt1513-nt1534) and 5’-CTTGAACAGTAGGACATGAACA-3’ (nt1847-nt1868). Cycling conditions for the initial amplification of nPCR consisted of 3 min pre-denaturation at 94°C; 30 sec denaturation at 94°C, 1 min annealing at 50°C and 2 min extension at 72°C for 35 cycles, and 7 min final extension at 72°C. The conditions for the second amplification consisted of 3 min pre-denaturation at 94°C; 20 sec denaturation at 94°C, 20 sec annealing at 50°C and 20 sec extension at 72°C for 35 cycles; and 7 min final extension at 72°C. The recombinant plasmid pUC18-HBV and sterile water were used as positive and negative controls, respectively. The amplification product was electrophoresed in 1.7% agarose gel. The 356 bp DNA band was purified and the sequence was confirmed by a commercial vendor (SinoGenoMax Co. Beijing, China).

### HBV-genotype analysis by nPCR

Analyses of HBV genotypes A through F were performed on the human sera which had been used as inocula and the sera obtained from infected tree shrews. The procedure as well as the sequences of genotype-specific primers have been previously published by Natio et al. [23]. The primers were synthesized by Sangon Biotech Co. (Shanghai, China). Briefly, the first round of PCR amplification employed universal outer primers for all of the six HBV genotypes, the products of this reaction were then used as template for the second round of PCR utilizing two different mixtures (A and B) of inner primers. Mix A consisted of a sense primer common for genotypes A, B and C, and three different antisense primers specific for genotypes A, B and C, respectively. Mix B consisted of an antisense primer common for genotypes D, E and F, and three different sense primers specific for genotypes D, E and F, respectively. The final products were subjected to gel electrophoresis.

### Detection of HBsAg and HBcAg by immunohistochemistry and histopathological examination on liver biopsies

Presences of HBsAg and HBcAg in paraffin-embedded liver biopsies were examined by Immunohistochemistry assays. Monoclonal antibodies of mouse anti-human HBsAg (Zhongshan Goldenbridge Biotech, Beijing, China) and mouse anti-human HBcAg (Maxim Bio, Fuzhou, China) were used in conjunction with UltraSensitive^TM^ S-P staining kit and DAB kit (Maixin Bio, Fuzhou, China), following the instructions provided by manufacturer. As positive control, HCC-surrounding liver tissue from HBV-infected HCC-patients was used.

Hematoxylin and eosin (HE)-stained liver biopsies were analyzed and interpreted by certified pathologists.

### Study specific criteria defining “confirmed” and “suspected” chronic-infection

For this study, we established the criterion for “confirmed chronic-infection” for which the same animal had to sustain detectable serum HBsAg in addition to either serum or liver detectable HBV DNA in excess of 48 weeks post inoculation. The criterion for “suspected chronic-infection” was the requirement for detectable HBV DNA over at least 48 weeks post inoculation, on at least two or more occasions. For “suspected chronic infection”, the HBsAb detection remained negative or was only occasionally positive during the early period following inoculation.

## Abbreviations

ELISA: Enzyme-linked immunosorbent assay; FQ-PCR: Fluorescence quantitative polymerase chain reaction; HBV: Hepatitis B virus; HBsAg: HBV surface antigen; HBsAb: HBV surface antibody; HBeAg: HBV e antigen; HBeAb: HBV e antibody; HBcAb: HBV core antibody; nPCR: Nested polymerase chain reaction; TRFIA: Time-resolved immunofluorescence analysis.

## Competing interests

Authors declare there are no financial nor non-financial competing interests in relation to this manuscript.

## Authors’ contributions

QW carried out the animal experiment and molecular studies, performed the statistical analysis, and participated in drafting the manuscript. PS made substantial contribution to interpretation of data, involved in revising manuscript and giving final approval of the version to be published. JJS, CY, JC and CO participated in study design, pathology and immunohistochemistry studies and animal experiment. FY, JJZ, LL JLS, FY (X), DPW, JW and XJW participated in the animal experiment and molecular studies. PR participated in the statistical analysis, pathology and immunohistochemistry studies. Yuan Li conceived of the study, carried out its design and helped to draft the manuscript. All authors read and approved the final manuscript.
